# Radio-frequency plasma polymerized biodegradable carrier for *in vivo* release of cis-platinum

**DOI:** 10.18632/oncotarget.10932

**Published:** 2016-07-29

**Authors:** Sudhir Bhatt, Fatemeh Valamanesh, Jerome Pulpytel, Rea Lo Dico, Aliby Baiyukha, Iman Al-dybiat, Marc Pocard, Farzaneh Arefi-Khonsari, Massoud Mirshahi

**Affiliations:** ^1^ Sorbonne Universités, UPMC Univ. Paris 6, CNRS, Laboratoire Interfaces et Systèmes Electrochimiques, 75005, Paris, France; ^2^ Sorbonne Paris Cité Universités, UMR Univ. Paris 7, INSERM U965 Carcinose, Angiogenèse et Recherche Translationnelle, L'Hôpital Lariboisière, 75010, Paris, France; ^3^ Current address: Frank Reidy Research Center for Bioelectrics, Old Dominion University, Norfolk, 23508, VA, USA

**Keywords:** plasma polymers, biodegradable coatings, *in vivo* drug delivery system, anti-cancer drug, targeted therapy

## Abstract

A low pressure plasma process based on plasma deposition has been used to develop a drug delivery strategy. In this study, a drug delivery system based on different layers of plasma co-polymerized Poly ε-caprolactone-Polyethylene glycol (PCL-PEG) co-polymers was deposited on biocompatible substrates. Cis-platinum (118 μgm/cm^2^) was used as an anti-cancer drug and incorporated for local delivery of the chemotherapeutic agent. The co-polymer layers and their interaction with cancer cells were analyzed by scanning electron microscopy. Our study showed that the plasma-PCL-PEG coated cellophane membranes, in which the drug, was included did not modify the flexibility and appearance of the membranes. This system was actively investigated as an alternative method of controlling localized delivery of drug *in vivo*. The loading of the anti-cancer drug was investigated by UV-VIS spectroscopy and its release from plasma deposited implants against BALB/c mice liver tissues were analyzed through histological examination and apoptosis by TUNEL assay. The histological examination of liver tissues revealed that when the plasma-modified membranes encapsulated the cis-platinum, the Glisson's capsule and liver parenchyma were damaged. In all cases, inflammatory tissues and fibrosis cells were observed in contact zones between the implant and the liver parenchyma. In conclusion, low pressure plasma deposited uniform nano-layers of the co-polymers can be used for controlled release of the drug *in vivo.*

## INTRODUCTION

In the past several years interest in platinum drugs has increased due to their successful administration for the treatment of most disseminated cancers [1]. Cis-platinum (cis-diaminedichloroplatinum (II)) remains one of the most widely used and potent anticancer agents against many different types of cancer [2–3]. Cis-platinum exerts its anticancer effects by covalently binding to DNA and forming various platinum-DNA adducts which cause distortions in DNA and that leads to final cellular outcome of apoptotic cell death [4]. For the cancer treatment, cis-platinum is usually intravenously administered but it is often unsuccessful because of the nonselective distribution of the drug among the normal and tumor tissue and that likely enhances the risk of dose limiting side effects including chronic neurotoxicity, acute nephrotoxicity and myelosuppression [5–6]. After 24 hrs of clinical administration of cis-platinum, it has been noticed that more than 65% of the platinum in the blood was protein bound, leading to severe side effects of cis-platinum treatment and therefore less therapeutic efficacy [7] and resistance over the course of medication [8]. In order to overcome these therapeutic limitations by cis-platinum treatment and other anticancer drugs, there is a clear incentive to develop new strategies for efficient, systematic and controlled release of cis-platinum for oncology.

Towards enhancement for the prevention of the systematic side effects of the chemotherapy, a wide range of strategies have been developed for a more selective delivery. The trend in drug delivery technology has been toward biodegradable polymer excipients requiring no follow-up surgical removal once the drug supply is depleted [9]. For instance, wet chemically prepared polymer based cis-platinum loaded drug delivery systems such as platinum encapsulated PLGA-b-PEG nanoparticles [10], liposomes [11], PEG-b-poly (amino acid)-based polymeric micelles [12], polymeric conjugates of γ-PGA–cis-platinum [13], pH sensitive Bi(PEG-PLA)-Pt(IV) polymer-prodrug conjugates [14] etc. The recent advances in nanoparticle formulations of cis-platinum for therapeutic applications have been thoroughly surveyed and the clinical utility of cis-platinum conjugates have been investigated [15].

As compared to the wet processes [9–16], plasma polymerization is a catalyst and solvent free, dry, one step, highly controllable and environmentally benign process. Recently, the radio frequency (RF) plasma (co-) polymerization of different organic precursors for surface modifications of a variety of substrates have been summarized in order to tailor the physico-chemical properties of the substrates for mitigation of nonspecific adsorption of proteins, tunable biomolecule-surface interactions and controlled drug delivery applications [17, 18]. The synergetic role of plasma generated free radicals for tuning the surface properties of polymers and their applications have been discussed broadly for drug delivery, biomedical and tissue engineering [19].

The biodegradable and biocompatible PCL-co-PEG polymers with different cell adhesion and cell-repellent properties have been prepared on different flat substrates by gradually varying the partial pressure ratio of the monomers (ε-CL/DEGME) under the solvent and catalyst free low-pressure inductively excited RF plasma reactor [20–23]. Later, cis-platinum loaded multilayer of plasma was studied polymerized PCL-co-PEG coatings have been used to produce barrier layers on different flat surfaces (glass, Si wafer, etc.) for the controlled release of drug and the *in vitro* release of anticancer drug was investigated for the apoptosis of ovarian cancer cells [22].

In our previous works [20–23], we have reported how to develop a Drug Delivery System based on multiple layers composed of plasma copolymers deposited on glass, and Si wafer by the dry clean plasma process. The controlled drug delivery of anticancer drug was investigated *in-vitro* for the apoptosis of ovarian cancer cells [22]. Here the same drug delivery system was deposited on cellophane membranes and the controlled release of cis-platinum was studied *in vivo*.

## RESULTS

### UV-visible spectroscopic measurements for cis-platinum loading

**Figure 1:**
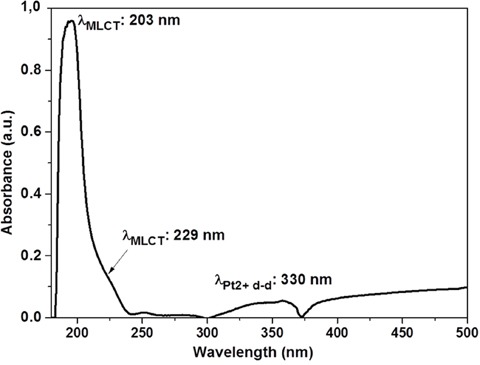
UV absorption spectrum of 1 mM Cis-platinum in 1X PBS at pH 7

The loading of cis-platinum on plasma co-polymerized cellophane substrates was determined by measuring the absorbance in the range of 200 – 500 nm using UV-Vis spectrometer. The absorption spectrum of 1mM cis-platinum in 1X PBS, pH 7, is shown in Figure 1. Because of metal to ligand charge transfer (MLCT) transitions in cis-platinum solution [23], a strong band at 203 nm and a shoulder at 229 nm are appeared. Cis-platinum compound exhibits a much weaker band at 330 nm which is assigned to the d-d transition of Pt^2+^ ions.

### Cell-membrane contact analysis by scanning electron microscopy

**Figure 2:**
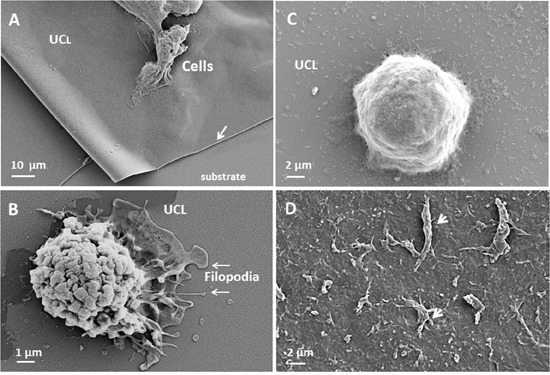
interaction of ovarian cancer cell with uniform copolymer layer (UCL) Ovarian cancer cell line OVCAR-3 adhered on the UCL (arrow) in the culture medium (**A.** X2500). This interaction is biocompatible and adhered cell form the pseudopod and nanopod on the UCL (**B.** X20000). When the anti-cancer drug was incorporated, on the UCL, the cells shape changed (**C.** x10000) and adherent necro-apoptotic cell organelles (arrow) were observed after cells detachments (**D.** X5000).

As presented in Figure 2A, uniform copolymer layer (UCL) is intact and posed correctly on the substrate. Ovarian cancer cell line OVCAR-3 adhered on the UCL in the cell culture condition and do not perturbed by culture medium. Adhered cells form the filopodias on the membrane (Figure 2B). Incorporation of cis-platinum strongly modified the physiological properties of UCL by induction of cells detachment (Figure 2C). As presented in Figure 2D, the cellular debris remained after cell death on the UCL.

### Influence of multilayer coatings membrane implants on the liver parenchyma

**Figure 3:**
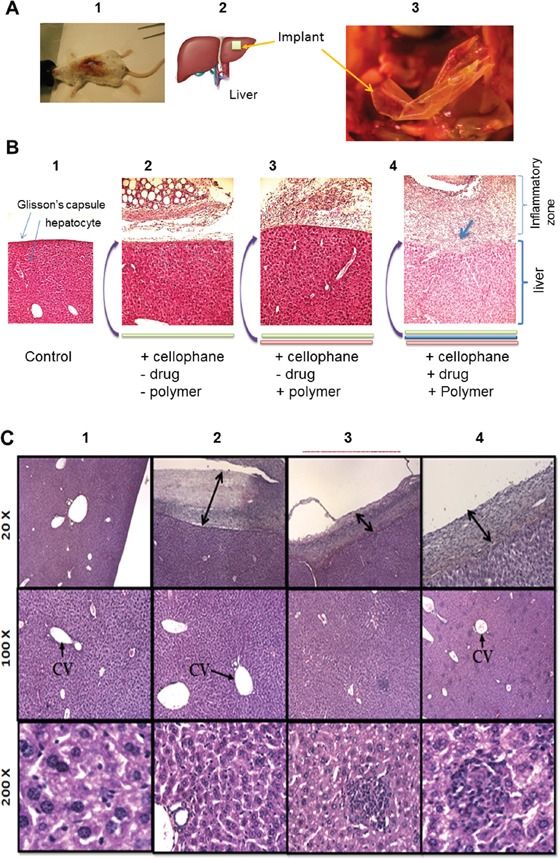
Macroscopic and microscopic observation of hepatic implantation of cellophane inBALB/c mice sacrificed on the 10^th^ day Macroscopic and representative image of cellophane implanted animal, magnified image showing surgically removed implant and representative image of histology **A**. Microscopic images for the histological analysis of normal liver tissues called of control **B-1**. liver exposed to untreated (uncoated) cellophane (***Cello***) **B-2**. plasma polymers + cellophane coated with copolymer without cis-platinum (4B-3) cellophane (*pPCL-co-PEG-Cello*) **B-3**. cellophane coated with copolymer + drug and plasma polymers, + Cis-platinum + cellophane (*Cisp-pPCL-co-PEG-Cello*) **B-4**. Data are a typical microscopic imaging of liver tissue sections **C.** under original magnification of 20 X, 100 X and 200 X. Nuclei: dark blue, cytoplasm: pink. CV: Central vein.

**Figure 4:**
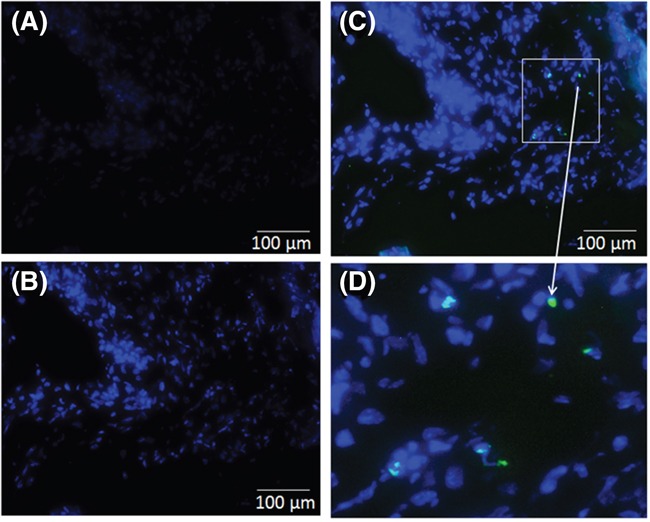
Apoptotic cells in liver tissue sections were detected using TUNEL assay for untreated cellophane (*Cello*) **A.** plasma polymers + cellophane (*pPCL-co-PEG-Cello*) **B.** plasma polymers + Cis-platinum + cellophane (*Cisp-pPCL-co-PEG-Cello*) **C.** and magnified image showing apoptotic cells **D.** Data are a typical fluorescent microscopic imaging of liver tissue sections under original magnification of × 100. Nuclei: blue, apoptosis cells: green.

In our previously published work [22], the cis-platinum loaded plasma polymerized multilayer PCL-co-PEG coatings was assessed *in vitro* using human ovarian carcinoma cells (NIH: OVCAR-3) where it has been demonstrated that controlled release of cis-platinum was effectively induced apoptosis. Herein, after 10 days of hepatic implantation, the mice liver was extracted by surgical resection and histological measurements were performed (Figure 3). In Figure 3A, 1-3, the macroscopic and representative image of cellophane implanted animal, the position of the implant on the liver, magnified image showing surgically removed implant and representative image of histology are presented. Figure 3B, 1-4, histological examination of plasma deposited implants wrapped onto a liver, showed that the implants were properly positioned and surrounded by a fibrous capsule. This capsule was homogeneous along the whole perimeter of the plasma coated implants, with limited areas of necrosis and inflammatory cells. Several liver sections from this region were analysed for each case (Figure 3B, 1-4). Normal (3B-1) and the liver exposed to bare cellophane, unloaded drug membranes were presented in Figure 3B, 1-3. The liver is invested by a connective tissue capsule named Glisson's capsule. The histological examination of the liver of the control mice exhibited the normal architecture of the liver whereas the parenchyma of the control liver specimens was uniform in appearance. As presented in Figures 3B-2, when untreated cellophane was used, the Glisson's capsule and liver parenchyma were intact after 10 days. In case of *pPCL-co-PEG-cello*, no significant change was observed in liver tissues as compared to control Figures 3B-3. That shows the plasma polymerized PCL-co-PEG coatings on cellophane implants which were in contact with liver for 10 days, were biocompatible and did not generate significant inflammatory response. The histological appearance of parenchyma in *pPCL-co-PEG-Cello*, and untreated cellophane was comparable with that of control group. On the contrary, when the membrane contained cis-platinum (*Cisp-pPCL-co-PEG-Cello*,), the Glisson's capsule and liver parenchyma were damaged (Figure 3B-4). In addition, the mice liver treated with the *Cisp-pPCL-co-PEG-Cello*, formulation did exhibit inflammatory cells as compared to the control. As compared to control, the histological examination of the liver tissues of the *Cisp-pPCL-co-PEG-Cello* treated mice exhibited congestion of the central veins and intracytoplasmic vacuolations were observed which might be due to the cis-platinum induced oxidative damage and lipid peroxidation of the liver tissues [24]. In the cells around the central vein (arrow CV), the cytoplasmic modifications were observed. As shown in Figure 3C (magnification: 200X), the clusters of inflammatory cells were observed in the surrounding of portal region. In all implanted cases, the fibrosis was observed on the zones in contact with the implant and liver parenchyma showed by arrow (Figure 3C, 2-4).

### Detection of apoptotic cells in liver tissues

In order to detect the apoptosis in the liver tissues, the TUNEL immunohistological assay was performed for different experimental groups (Figure 4). As shown in stained sections (Figure 4A and 4B), no significant morphological differences were observed in liver tissues from mice implanted with untreated cellophane (Figure 4A) and plasma treated cellophane (Figure 4B). No apoptotic cells were observed with untreated cellophane (*Cello*) and *pPCL-co-PEG-Cello*. TUNEL positive (green florescence) signals were, however observed in liver tissues of mice wrapped with *Cisp-pPCL-co-PEG-Cello* implants for 10 days (Figure 4C). The magnified image of TUNEL positive cells in liver tissues is shown in Figure 4D.

### Cis-platinum traces in blood plasma by ultraviolet-visible spectrophotometry

**Figure 5:**
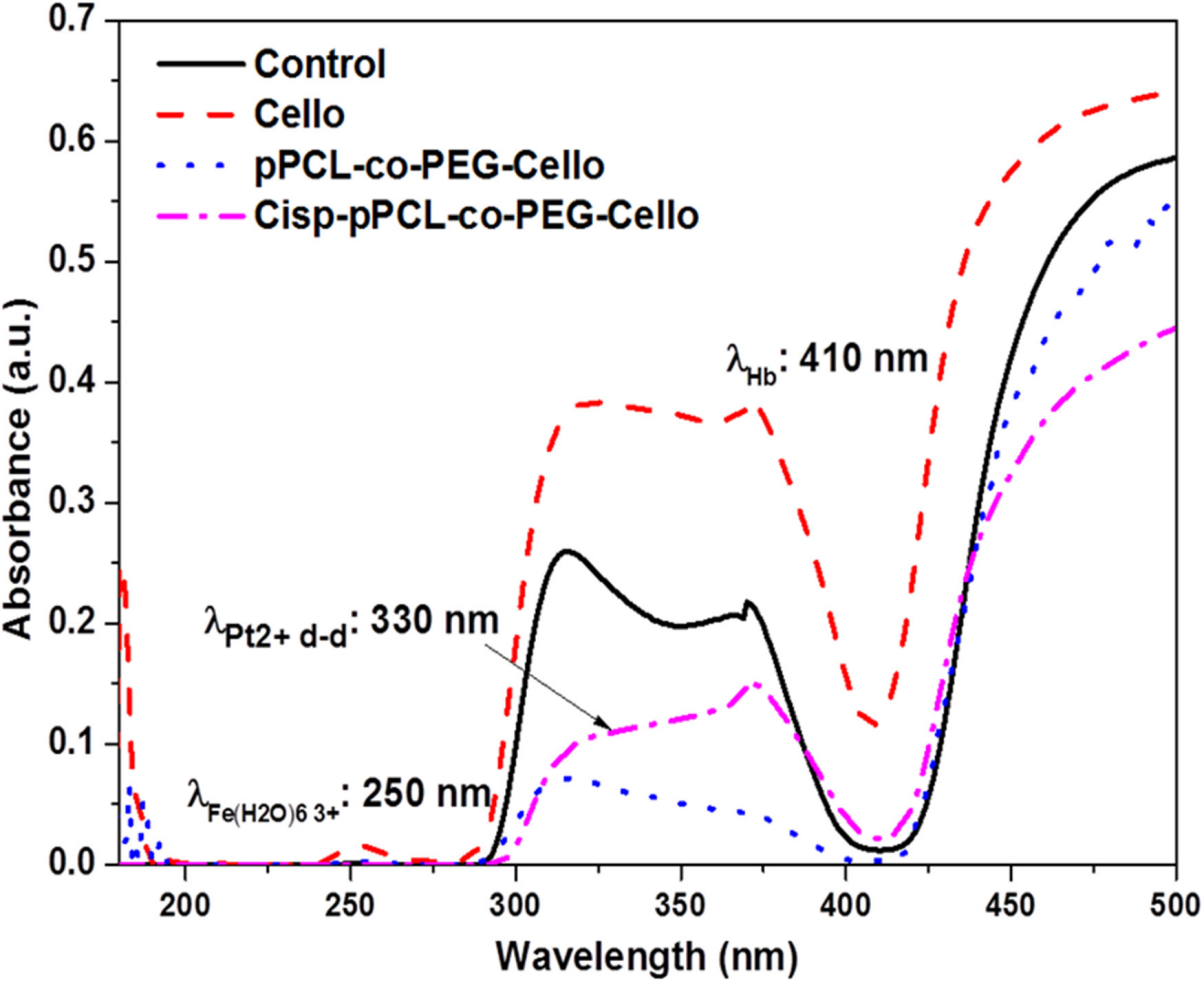
UV absorption spectrum of blood plasma for control, untreated cellophane (*Cello*), plasma polymers + cellophane (*pPCL-co-PEG-Cello*), and plasma polymers + Cis-platinum + cellophane (*Cisp-pPCL-co-PEG-Cello*)

Preliminary studies were carried out to determine the traces of cis-platinum in the blood plasma in the broad ultraviolet-visible (UV-Vis) region ranging from 200 to 500 nm. Initial studies were conducted using 0.2-0.5 ml of blood plasma from experimental groups and control group in a quartz cuvette. As presented in Figure 5, The UV-V of the spectrum of the blood plasma shows a strong band at 410 nm and a less intense band at 250 nm attributed to the hemoglobin (Hb) and oxidized Hb respectively. In the case of *Cisp-pPCL-co-PEG-Cello*, a very weak peak corresponding to Pt^+2^ ion can be identified at 330 nm, even though it is not completely distinguishable from the rest of the spectra due to the overlapping. Therefore it is difficult to assign the absorption of platinum compounds in biological medium such as blood. One can note in Figure 2 which shows the UV absorption of cis-platinum in PBS, (Figure 5). It can be pointed out that the absorption spectrum of cis-platinum in blood plasma (Figure 5) is quite different as compared to its absorption in PBS (Figure 2). Therefore it is difficult to assign absorption of platinum compounds in biological media such as blood.

## DISCUSSION

**Figure 6:**
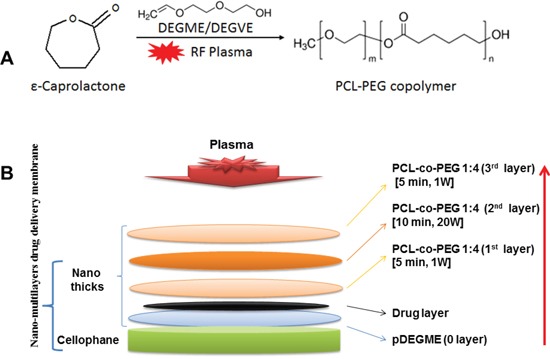
Schematic of plasma polymerized multilayer coatings on cellophane for *in vivo* release of cis-platinum **A.** Radio-Frequency Plasma Polymerization (RF-Plasma) of ε-Caprolactone and DEGDME for generation of PCL-PEG copolymer on the membrane (cellophane). **B.** Schematic of the multi-stack PCL-PEG coating for a tuneable release of entrapped drug layer (cis-platinum).

In this study we described the *in vivo* local drug release efficacy of the low pressure plasma polymerized PCL-co-PEG encapsulated cis-platinum implants as biocompatible and biodegradable drug carriers. A drug delivery system based on different layers presenting different chemical compositions of PCL-PEC copolymers obtained by low pressure plasma has been already reported [22]. The total thickness of these layers is in the order of a few hundreds of nm. However the first layers deposited to increase the affinity of the membrane is a few tens of nm, followed by a dense barrier deposited at higher powers, covered again by coatings under conditions which would be cell nonadhesive (Figure 6B). Indeed the reason why we have used PCL-PEG(1:4) is because the copolymers deposited under such conditions when deposited at low power can be cell nonadhesive while at higher powers they are dense and crosslinked and act as a barrier for leaching out of the drugs. In this work such multi-layer coatings have been deposited on biocompatible cellophane substrates which were flat, non-porous surfaces (2D). Cis-platinum loaded (118 μgm/cm^2^) PCL-co-PEG coatings with optimized ratios of ε-CL/DEGME monomer feed in the plasma reactor were prepared for controlled cell death applications. The plasma treatments did not modify the flexibility and appearance of the membranes. As demonstrated by SEM, the copolymer layer was identifiable and the cancer cells forming the pods adhered well on the copolymer. These results suggest the biocompatibility and non-cytotoxicity of the membrane complex in the absence of drug. After incorporation of the drug, the membrane becomes cytotoxic. Cis-platinum-loaded cellophane implants were placed directly at the site of mice liver, offering a controlled and local release of drug by means of polymer swelling and/or dissolution and/or disintegration. The UV-Vis spectroscopic measurements were conducted on both cis-platinum solution in PBS (drug loading) and cis-platinum release in blood plasma. By using UV absorption measurements, we showed that cis-platinum reacted with proteins in the blood plasma and a derivatization technique is required for the quantification of cis-platinum in blood stream. By the different biochemical techniques, the reaction abilities of the platinum complexes such as cis-platinum to blood serum proteins have been reported before [25]. As compared to other proteins such as albumin and immunoglobulin, higher reaction (binding) ability of cis-platinum to hemoglobin (Hb) has been shown [26]. In our case of cis-platinum in blood plasma, a high absorbance of Hb was obtained at 410 nm as compared to the intensity of the cis-platinum for Pt^+2^ at 330 nm. After 10 days of implantation, the detection of traces of cis-platinum in the blood plasma might be difficult by means of UV absorption measurements without carrying out derivatization which result from the *in vivo* formation of cis-platinum-protein adducts via strong interactions between blood plasma and platinum ligands. Due to the complexity of the blood plasma and the low molecular absorptivity of cis-platinum in the UV region, the selective derivatization for the detection of cis-platinum in biological samples has been discussed by means of optical measurements of the platinum-ligand drugs [27].

Our previous *in vitro* study using plasma polymerized PCL-co-PEG coated cis-platinum for controlled release has confirmed the cytotoxic effects of cis-platinum to cancer cells [22].

Then the cellulose based polymer was covered with the multi-layered coating according to the procedure explained and was implanted on Glisson's capsule of the liver. The *in vivo* measurements showed that all animals reacted against the implant and after 10 days of post-implantation, inflammatory tissues were generated in the region of implants. Consequently, macroscopic and microscopic analysis demonstrated endogenous tissues containing the micro-vessels, fibrocytes, immune inflammatory cells, tissues matrix and fibrosis. In all experiments except when the implant contained the drug, Glisson's capsule was intact. In contrast, when implants contained the drug, the release induced the rupture of the Glisson's capsule and consequently, the liver parenchyma was destroyed. The presence of apoptotic cells only in liver parenchyma in the presence of the implant which encapsulated the drug suggested the efficacy of nano thick multilayer drug delivery membrane. These implants provided the release of high amounts of the drug without any complication in surrounding organs stomach, spleen, diaphragm, and others digestive organs including renal toxicity (Supplementary Results, Figure S-1). All the 20 mice were alive and healthy on Day 10.

In the present work, the histological examination of the liver tissues which were in contact with cis-platinum loaded plasma polymerized cellophane implants were showed hepatocellular vacuolization and cis-platinum induced inflammation of cells. As compared to untreated cellophane implanted on liver tissues, the apoptotic (TUNEL positive) cells were remained in the liver tissues which were in contact with cis-platinum loaded implants. Herein, we have shown the advantage of the one step, dry, clean, catalyst and solvent free soft plasma polymerized drug delivery system which can be prepared on a wide range of substrates and to achieve high purity materials and interfaces for *in vitro* and *in vivo* biological and clinical applications. In previous works, we used a wet chemical targeting method for ganciclovir-loaded albumin nanoparticles for intra ocular injection [28]. Co polymerization of the materials destined for nanoparticles generation, needed catalyser and solvent. Here, compared with traditional methods, we proposed plasma process which allows copolymerizing by means of a catalyst and solvent free process to regulate the drug release from a substrate.

However, we need further investigation to demonstrate the exact mechanism for loading and controlled release of cis-platinum induced hepatotoxicity and dose dependent effects on cancer tissues. In conclusion, low pressure plasma coating technique may be used to deposit coatings of uniform thicknesses over a variety of substrates for biological and clinical applications.

## MATERIALS AND METHODS

### Animals

A total of 20 female BALB/c mice (4 weeks old) were purchased from Charles River Laboratories (F 69592 L'Arbresle) France and body weight ranging from 20 to 30 g. All animals were maintained at the animal center for 2 weeks of adaptive feeding prior the start of experiment. Animals were randomly divided into three experimental groups and a control group. The mice were caged in groups of five in an air-filtered laminar flow cabinet and fed with irradiated food and autoclaved reverse-osmosis treated water. All procedures were performed under sterile conditions in a laminar flow hood. Five animals for each group were used. The experimental protocol was approved by the Ethics Review Committee for Animal Experimentation of UPMC, France. All experimental protocols were performed in accordance with the European Convention for the protection of vertebrate animals used for experimental and other scientific purposes (Council of Europe, 1986, ETS No. 123).

### Materials

ε-CL (Purity: 97%, MW: 114, Empirical formula: C_6_H_10_O_2_) and DEGME (Purity: 99.5%, MW: 134.17, linear formula: (CH_3_OCH_2_CH_2_)_2_O) were purchased from Sigma Aldrich, France and used in this study without further purifications. For the drug delivery implant applications, a 50 μm thick sheet of Cellulose bio-polymer was purchased from Humeau laboratories France. Cellophane substrates were cut to sizes of approximately 2 cm^2^ for all experiments.

### Antineoplastic drug

Cisplatin or cis-diamminedichloroplatinum (II) (CDDP) (linear formula: PtCl_2_H_6_N_2_, MW: 300.05) was used as an antineoplastic (anticancer) drug in sterile aqueous solution. Each mL of cisplatin solution, was used for the current study contained 1 mg of cisplatin and 9 mg sodium chloride in water (pH 3.5-4.5). For the *in vivo* drug release study, 20 μl of cisplatin solution of 1 mM concentration in 1X PBS was dropped onto cellophane substrates pre-coated with plasma polymerized PEG (pDEGME) and allowed to dry at room temperature. The cellophane slides (2 cm2) loaded with cisplatin (118 μgm/cm2) were immersed in 1 mL of 1X PBS (pH 7.0) at 37°C. The sample was agitated for 5 minute with a vortex mixer, then sonicated (Ultrasonic Cleaner, Fisher Scientific, France) in a room temperature water bath for 5 minute, and mixed with a vortex mixer for an additional minute. Afterwards, the supernatant was collected and the cisplatin loading was determined by UV Visible spectroscopic (SAFAS UVmc2, France) measurements. Spectral data was collected from 180-500 nm, (Supplementary Results, Figure S-2).

### Plasma polymerization of PCL-co-PEG coatings

PCL and PEG (PCL-co-PEG) copolymer coatings were fabricated in a low pressure inductively excited radio frequency-tubular quartz plasma reactor system (5 cm diameter, 40 cm length, base pressure of 3×10^−2^ mbar). The schematics of plasma deposition setup for PCL-PEG coatings, technical details of the process and *in vitro* characterization of cis-platinum release as well as the cell-surface interactions have been provided in our earlier work [26]. Briefly, prior to each experimental run, the reactor was scrubbed and cleaned with detergent, organic solvents and dried using compressed air. After cleaning with solvent, the plasma reactor was reassembled and cleaned further with 30W argon plasma discharge at 0.5 mbar pressure for 30 min. The partial pressure ratio of the two monomer fed in the reactor was controlled by the flow rate of carrier gas (i.e. Ar), which was regulated and measured by electronic mass flow controllers (MKS instruments). The flow rate of carrier gas was varied from 5 to 20 sccm with the increment of 5 sccm which was denoted by “1” for 5 sccm and “4” for 20 sccm respectively. The total flow rate was varied from 5 sccm to 25 sccm by keeping the operating pressure constant. The power was generated by Dressler Cesar RF generator which was delivered through the L-C matching network. The substrate is placed 9.0 cm below the coil. In the present work, we have deposited copolymer coatings for barrier layer at 20W CW (Continuous Wave) plasma which was then followed by deposition of a copolymer in a 1W PW (Pulse Wave). For the pulsed plasma discharge, the peak power (P_pk_) was 25W and the duty cycle (DC = (t_on_/(t_on_+t_off_), where t_on_ and t_off_ were the ‘plasma ON’ and ‘plasma OFF’ times respectively) was 4% to obtain the effective plasma power (P_eff_) which was 1W PW (t_on_ = 4ms and t_off_ = 96ms). Plasma polymerization of organic monomers was carried out on biocompatible cellophane substrates. All the coatings were deposited for different time duration (t_d_). After polymer deposition, the reactor was again evacuated to base pressure before the plasma polymerization system was vented to atmospheric pressure with air.

### Determination of cis-platinum loading

The cis-platinum solution was dispersed on pDEGME deposited cellophane substrates. In order to ensure cis-platinum recovery occurred from the cellophane implants, one implant was then cut into four individual pieces and submerged in 1 mL of 1X PBS (pH 7.0). The sample was agitated for 5 minute with a vortex mixer, then sonicated (Ultrasonic Cleaner, Fisher Scientific) in a room temperature water bath for 5 minute, and mixed with a vortex mixer for an additional minute. Afterwards, the supernatant was collected and the cis-platinum loading was determined by UV Visible spectroscopic (SAFAS UVmc^2^, France) measurements. Spectral data was collected from 180-500 nm.

### Scanning electron microscopy

10^5^/10μl of Suspended ovarian cancer cell line OVCAR-3 were deposited on a plasma polymerized glass and incubated for one minute then 2ml of RPMI medium (with 10% of Fetal Bovine Serum FBS, 1% of L-Glutamine and 1% of Streptomycin) were added to the well. All the samples were incubated at 37°C and 5% CO_2_ for 48h. The culture medium was discarded and samples were washed. The samples were fixed in 2% glutaraldehyde and 1X phosphate buffer saline (PBS, pH 7.4) at room temperature for 1 hour, washed in 1X PBS and then post fixed in 1% osmium-1X PBS for 45 min at room temperature and in dark conditions. After a final wash in 1X PBS, the samples were dehydrated in increasing concentrations of ethanol. Samples were dried by the critical point method with liquid CO2 and then sputter-coated with gold. They were observed with a S260 CAMBRIDGE scanning electron equipped with a LaB_6_ filament operating at 15kV and images were captured with the software “Orion” from (NCH Software).

### Preparation of implants

A schematic of plasma deposited multilayer PCL-co-PEG coatings on cis-platinum loaded cellophane for hepatic implantation is illustrated in Figure 6. For *in vivo* release of cis-platinum, the multilayer of plasma polymerized PCL-PEG copolymers were prepared on cellophane substrates as a multistep process discussed as follows: (i) 0^th^ layer: a hydrophilic layer of plasma polymerized DEGME (pDEGME) coating was deposited on 2 cm^2^ cellophane substrates for 5 min at 1W effective power. (ii) Cis-platinum (118 μgm/cm^2^) solution was mobilized on pDEGME coated cellophane substrates. (iii) 1^st^ layer: To avoid the direct exposure of the RF plasma, PCL-co-PEG (1:4) coatings were deposited on cis-platinum loaded cellophane substrates under mild conditions (P_pk_=25 W, DC=4%, P_eff_=1 W, t_d_=5 min). (iv) 2^nd^ layer: The barrier layer coatings were deposited to encapsulate the drug under 20 W CW power for 10 min. (v) 3^rd^ layer: Finally, PCL-co-PEG (1:4) coatings were deposited on top of the barrier layer under mild conditions for controlled cell-surface interactions (Figure 6). All the plasma deposited implants were prepared 1 hr before the *in vivo* experiments. All the experimental conditions for preparation of implants are summarized in Table 1.

**Table 1:** Experimental conditions for preparation of drug delivery vehicles for *in vivo* implants

*Implant*	*Description*	*Cellophane*	*pDEGME (0^th^ layer)*	*Cisplatin loading*	*Protective coating (1^st^ layer)*	*Barrier coating (2^nd^ layer)*	*Functionalized coating (3^rd^ layer)*	*No of mice per group*
***Control***		***Χ***	***Χ***	***Χ***	***Χ***	***Χ***	***Χ***	***5***
***Cello***	***Untreated Cellophane***	***√***	***Χ***	***Χ***	***Χ***	***Χ***	***Χ***	***5***
***pPCL-co-PEG-Cello***	***Cellophane +Plasma Polymers***	***√***	***√***	***Χ***	***√***	***√***	***√***	***5***
***Cisp-pPCL-co-PEG-Cello***	***Cellophane +Cisplatin +Plasma Polymers***	***√***	***√***	***√***	***√***	***√***	***√***	***5***

### Membrane implantation

All 20 animals were anesthetized with 2% isoflurane in oxygen with mechanical ventilation for 15 min. Each animal was fixed in a supine position. After skin disinfection with Betadine, a xifo-umbilical laparotomy was performed. A verification of the peritoneum and the abdominal cavity was realized and a self-retaining retractor was placed. If necessary, an Allis clamp type was placed on the xiphoid cartilage to improve exposure. The implants of cellophane (2 cm^2^) were placed above the left hepatic lobe, between the liver and the anterior face of the stomach. Untreated (*Cello*), plasma polymers (*pPCL-co-PEG-Cello*) and plasma polymers + Cis-platinum (*Cisp-pPCL-co-PEG-Cello*) treated cellophane were implanted into animals in the three different experimental groups (Table 1). In the control group, all animals were remained as received. The absorbable compress Surgicel® (Oxidized regenerated cellulose) was used to completely cover the implant and avoid the displacement (migration). The peritoneum was closed with a continuous suture of Prolene 7/0 (Ethicon, Somerville, NJ) a non-absorbable over lock or metal clips was placed on the skin. After surgical procedure, the anesthetics were discontinued, and all animals were allowed to recover for 20 minutes in a box flushed with 100% oxygen and then placed in their home cages. After 10 days from implantation, all mice from both experimental and control groups were euthanized by CO_2_ inhalation. General conditions of the surrounding tissue and the implanted cellophane substrates were observed with the naked eye. The part of liver opposite to implant was removed, fixed in PAF (4%) and embedded in paraffin. The slides (4 micron) were produced and colored by hematein-eosin-safran (HES) according to classical methods in the anatomo-pathogical laboratory. Histopathological study of liver tissues was performed

### Terminal deoxynucleotidyl transferase dUTP nick-end labeling (TUNEL) assay

After 10 days of implantation, the apoptotic cells in the liver tissues adjacent to the cellophane implants were detected using TUNEL (Promega, Madison, WI) assay. In brief, the sections of liver tissues were deparaffinined and dehydrated, followed by permeabilization with 20 μg/mL proteinase K and 0.2% Triton X-100 in 1X PBS. The slides were then labeled with a TdT reaction mixture for 90 min and were mounted with a mounting solution containing 4′, 6-diamidino-2-phenylindole (DAPI). The apoptotic cells (green) and cell nucleus (blue) was examined using a fluorescence microscopy.

### *In vivo* release of cis-platinum

The blood samples were collected from the mice retro orbital sinus into Heparin tubes after 10 days of implantation. The sampled blood (0.3-0.5 mL) were centrifuged at 3000 rpm for 10 min at 4°C to separate the plasma. Cis-platinum traces in the blood plasma was investigated using UV Vis Spectroscopic (SAFAS UV mc^2^, France) analysis. Spectral data was collected from 170-500 nm.

## SUPPLEMENTARY FIGURES


