# Supplementary material for: Radio-frequency plasma polymerized biodegradable carrier for in vivo release of cis-platinum
Source: Oncotarget. 2016 Jul 29;7(36):58121–32. doi: 10.18632/oncotarget.10932 (PMC5295417; doi:10.18632/oncotarget.10932)
Supplement: Supplementary file 1 [file oncotarget-07-58121-s001.pdf]

Radio-frequency plasma polymerized biodegradable carrier for *in vivo* release of cis-platinum

SUPPLEMENTARY FIGURES

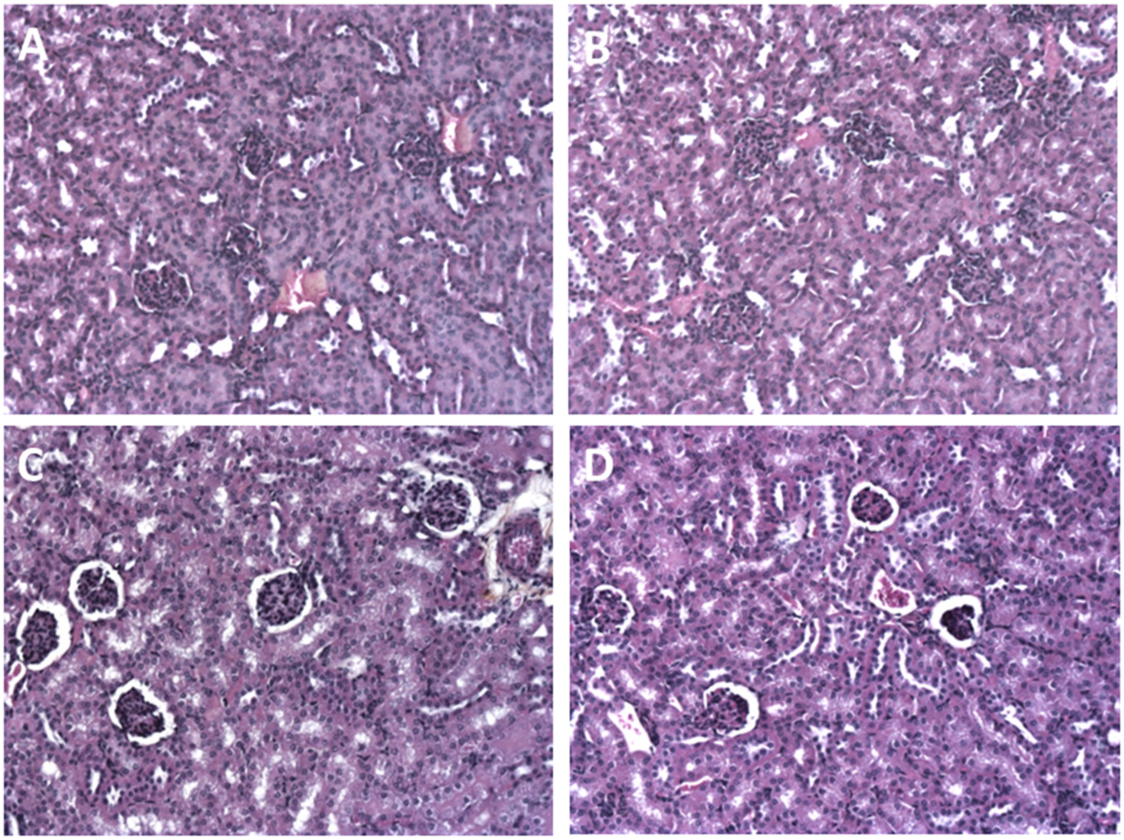

Supplementary Figure S1: Anathomopathological analysis of kidney. Several kidney sections were analysed by microscopy for each case. Various regions of the uriniferous tubule consisting the nephron and the collecting tubule in cortical labyrinth, medullary ray and medulla were explored. As presented in figure, no difference was observed when the control **A.** was compared with untreated implant **B.** plasma-polymer treated implant **C.** and drug containing **D.**

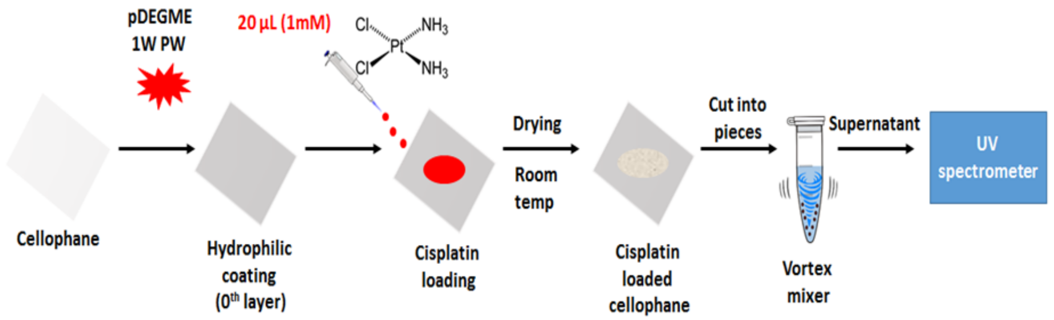

Supplementary Figure S2: UV measurements of Cisplatin loading and recovery. The cellophane slides pre-coated with plasma polymerized PEG (pDEGME) loaded with cisplatin and allowed to dry at room temperature, The sample was agitated for 5 minute with a vortex mixer, then sonicated in a room temperature water bath for 5 minute, and mixed with a vortex mixer for an additional minute. Afterwards, the supernatant was collected and the cisplatin loading was determined by UV Visible spectroscopic (SAFAS UVmc2, France) measurements. Spectral data was collected from 180-500 nm.
